# Regulation of sphingolipid metabolism in the immune microenvironment of gastric cancer: current insights and future directions

**DOI:** 10.3389/fonc.2025.1604227

**Published:** 2025-07-31

**Authors:** Yunqi Hua, Gangling Zhang, Yubo Liu, Xiaoling Tian, Xinyi Zhang, Ge Song, Qinggang Tian, Fangrui Yin

**Affiliations:** ^1^ Department of Medical Oncology, Baotou Cancer Hospital, Baotou, Inner Mongolia, China; ^2^ Department of Graduate School, Baotou Medical College, Inner Mongolia University of Science and Technology, Baotou, Inner Mongolia, China; ^3^ Department of Rheumatology, The First Affiliated Hospital of Baotou Medical College, Baotou, Inner Mongolia, China

**Keywords:** sphingolipid metabolism, gastric cancer, immune microenvironment, regulatory role, mechanistic research

## Abstract

In recent years, the role of sphingolipid metabolism in the tumor immune microenvironment has gradually gained attention, particularly in gastric cancer research. Sphingolipids are crucial components of cell membranes that regulate cell signaling and immune responses, making them important in tumor biology research. Despite numerous studies exploring the relationship between sphingolipid metabolism and gastric cancer, the specific regulatory mechanisms remain unclear. Further investigation is needed to understand their roles in the immune microenvironment. This article aims to review the regulatory mechanisms of sphingolipid metabolism in the immune microenvironment of gastric cancer, discussing its potential applications in tumor occurrence, development, and treatment. By analyzing current research progress, we will clarify the complex relationship between sphingolipid metabolism and immune cell interactions and look forward to future research directions, hoping to provide new ideas and strategies for immunotherapy in gastric cancer.

## 1 Introduction

Gastric cancer is a common malignant tumor worldwide, with varying incidence and mortality rates across regions. Epidemiological studies indicate that gastric cancer is particularly prevalent in East Asian countries, especially China and Japan. This prevalence is associated with dietary habits, Helicobacter pylori infections, and genetic factors (1). Although there have been advancements in early screening and treatment technologies for gastric cancer in recent years, clinical challenges remain significant, primarily manifested in late diagnosis, difficulties in treatment plan selection, and drug resistance (2). Therefore, a deeper understanding of the mechanisms underlying gastric cancer, especially how the tumor microenvironment plays a role, is crucial for improving patient prognosis.

The immune microenvironment plays a very important role in tumor occurrence. It not only affects the growth and metastasis of tumor cells but also plays a key role in the immune evasion mechanisms of tumors. Studies have shown that immune cells, cytokines, and extracellular matrix components in the tumor microenvironment collectively shape the biological characteristics of tumors (3). Particularly in gastric cancer, how the infiltration of tumor-associated macrophages and regulatory T cells relates to patient prognosis suggests the importance of considering the modulation of the immune microenvironment in treatment strategies.

Sphingolipid metabolism is a crucial aspect of cell biology that includes the synthesis and breakdown of various bioactive molecules. Sphingolipids are essential components of cell membranes and contribute to cell signaling, growth, and apoptosis (4). Recent studies have revealed a strong link between sphingolipid metabolism and the tumor immune microenvironment. Metabolic products of sphingolipids can influence the function and activity of immune cells, playing significant roles in tumor formation and progression (5). This correlation opens up new research directions. It suggests that by modulating sphingolipid metabolism, we can improve the tumor immune microenvironment and enhance treatment outcomes for gastric cancer patients.

## 2 Classification and metabolic pathways of sphingolipids

### 2.1 Main types of sphingolipids and their biosynthesis

**Figure 1:**
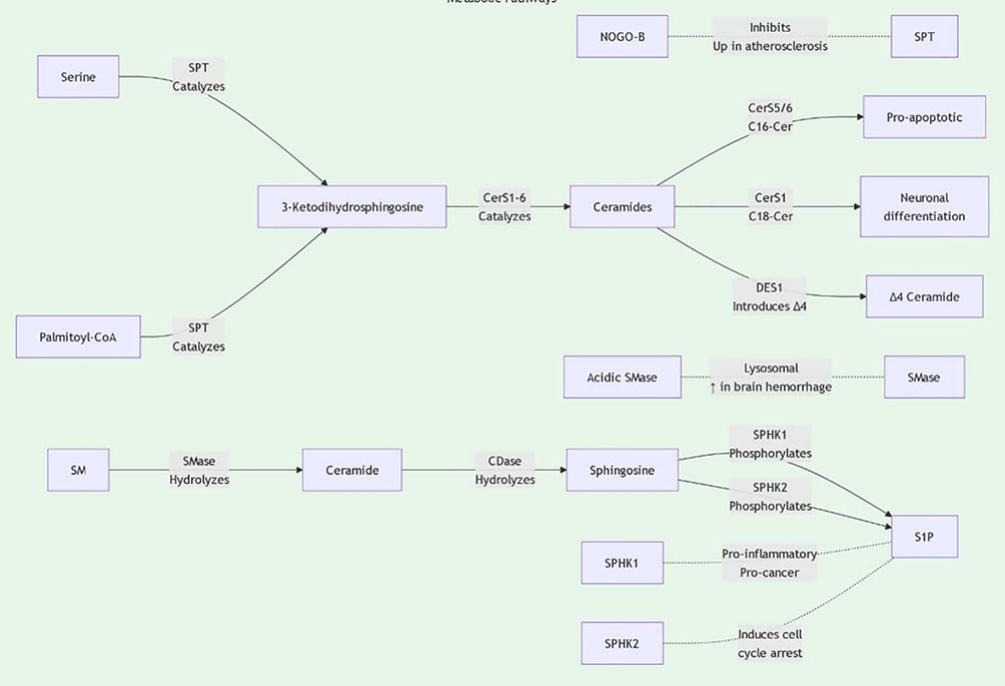
Sphingolipid metabolic pathway diagram1.

**Figure 2:**
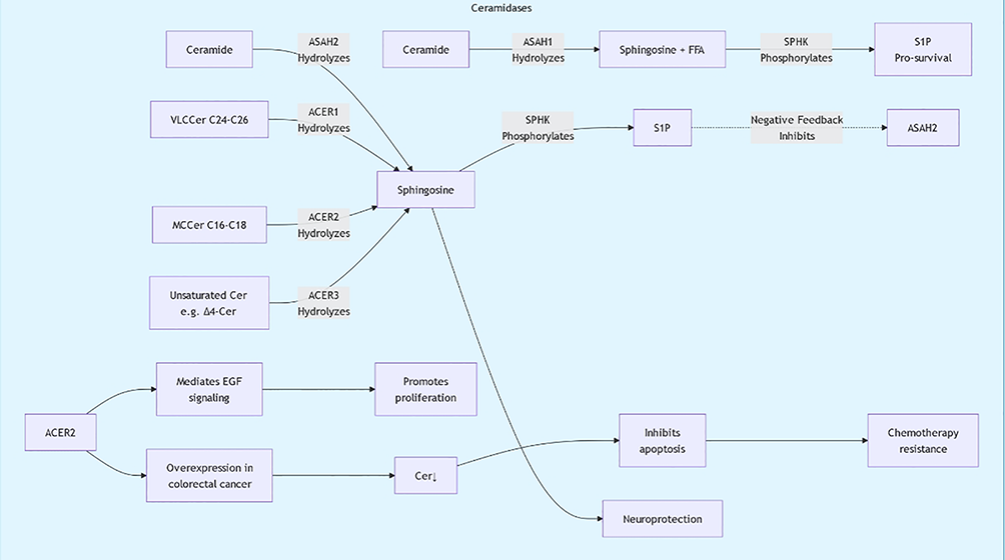
Sphingolipid metabolic pathway diagram2.

Sphingolipids are essential for the structure and signaling of cell membranes. Sphingolipids can be classified into several main types based on their structure and function: ceramides, sphingomyelins, glycosphingolipids, and polysaccharide sphingolipids. Ceramides, the core components of sphingolipids, are typically formed by combining long-chain fatty acids with amino alcohols. Sphingomyelins are formed by combining ceramides with phosphate and either choline or ethanolamine. These molecules are widely present in cell membranes, particularly in the nervous system. Glycosphingolipids are formed by the combination of sugar molecules with ceramides, primarily involved in cell recognition and signal transduction. Sphingolipid biosynthesis primarily occurs through the classical acylation-deamination process. This process involves multiple enzymes, including ceramide synthases. The activity of these enzymes is regulated by various factors, including the lipid environment within the cell and external signals (**Figure 1**, **2**). For example, studies have shown that ceramide synthesis may be upregulated in tumor cells, promoting tumor growth and metastasis (6, 7).

#### 2.1.1 Sphingomyelin

Sphingomyelin is an essential type of lipid predominantly located in cell membranes, particularly in nervous tissue. Its basic structure consists of a sphingosine backbone linked to a phosphate group, which can form derivatives like sphingomyelin choline and sphingomyelin ethanolamine. Research indicates that sphingomyelin is crucial for cell signaling, maintaining membrane structure, and facilitating intercellular interactions. Recent studies have found that issues related to sphingomyelin metabolism are linked to several diseases, including Alzheimer’s disease and cardiovascular conditions (8, 9). Furthermore, changes in sphingomyelin may act as biomarkers for certain cancers; for example, specific sphingomyelin profiles in advanced colorectal cancer patients can be used as prognostic indicators (7). Therefore, understanding the biological functions of sphingomyelin and its involvement in diseases is vital for the development of new therapeutic strategies.

#### 2.1.2 Glycosphingolipids

Glycosphingolipids are lipids composed of a sphingosine and one or more sugar molecules. They form microdomains on cell membranes, interacting with membrane proteins and participating in cell recognition and signal transduction. Research shows that glycosphingolipids play a key role in immune responses and neurodevelopment. For example, certain types of glycosphingolipids are important in the activation of macrophages, influencing the intensity and duration of inflammatory responses (10). Additionally, changes in glycosphingolipids are associated with the occurrence of autoimmune diseases such as rheumatoid arthritis, with studies finding significantly elevated levels of monosaccharide sphingolipids in the serum of rheumatoid arthritis patients (11). Therefore, glycosphingolipids are not only components of cell membranes but also play important roles in various physiological and pathological processes.

#### 2.1.3 Sphingosine

Sphingosine is the fundamental component of sphingolipids, with various biological functions. Sphingosine is an important precursor for sphingolipid synthesis. Additionally, it participates in cell signaling and regulates processes like cell growth, proliferation, and apoptosis. Studies show that sphingosine metabolic products, such as sphingosine-1-phosphate, promote cell proliferation and survival, especially in cancer cells (12). Furthermore, dysregulation of sphingosine metabolism is closely linked to various diseases, including metabolic syndrome, cardiovascular diseases, and neurodegenerative disorders (13). Therefore, studying sphingosine metabolism and its roles in diseases enhances our understanding of its physiological functions and offers new insights for treating related conditions.

#### 2.1.4 Regulatory mechanisms of sphingolipid metabolism

The biosynthesis of sphingolipids primarily occurs through the sphingosine pathway. Sphingosine synthesis starts with amino acid metabolism. This is followed by several enzymatic reactions that convert sphingosine into various types of sphingolipids. Recent studies have revealed the importance of sphingolipid metabolism in cellular functions and disease progression. Abnormal sphingolipid metabolism is closely linked to various cancers, especially within the tumor microenvironment, where alterations in sphingolipids can influence tumor cell growth and metastasis (12, 14).

The synthesis of sphingolipids is primarily carried out by ceramide synthases, involving the cooperative action of multiple key enzymes. These enzymes combine fatty acids with acyl-CoA to generate sphingosine, which is further converted into sphingolipids. This process is influenced not only by substrate availability but also by the regulation of intracellular signaling pathways. Studies have shown that vitamin K2 can regulate the *de novo* synthesis pathway of sphingolipids, thereby affecting cellular physiological functions and overall metabolism (15). Additionally, the synthesis of sphingolipids is also related to cellular stress responses, which in certain cases can lead to accelerated apoptosis, a phenomenon particularly important in cancer research (16).

Sphingolipids mainly degrade through hydrolysis and oxidation, facilitated by enzymes like sphingomyelinases and sphingosine kinases. These enzymes break down sphingolipids into sphingosine and fatty acids, which can then convert into sphingosine-1-phosphate, a key participant in cell signaling. The degradation products of sphingolipids play important roles in cell migration and proliferation, influencing tumor metastasis and prognosis (6).

**Figure 3:**
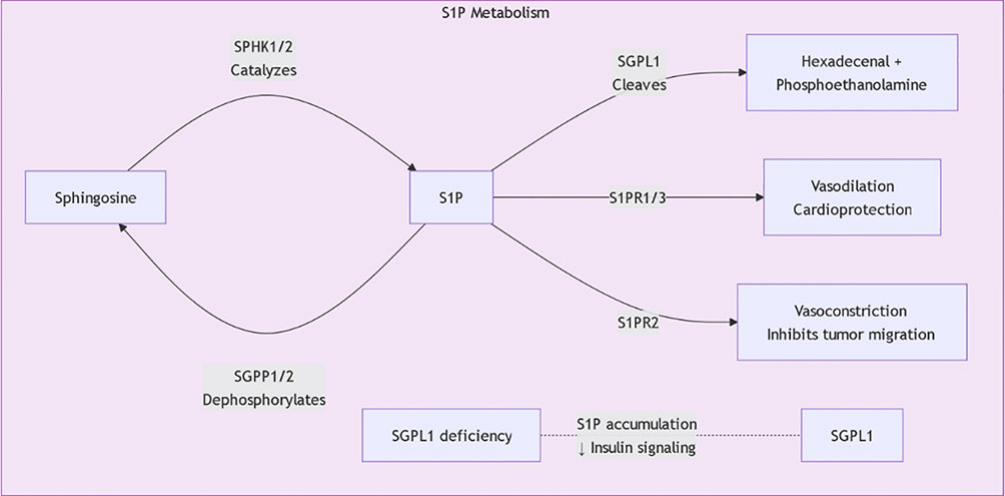
S1P metabolic pathway.

Metabolic products of sphingolipids, such as sphingosine and ceramides, have been shown to affect the progression of the cell cycle, thereby influencing the proliferation and survival of tumor cells. For instance, research shows that alterations in sphingolipid metabolism can affect the expression of proteins related to the cell cycle, which in turn influences cell proliferation. Specifically, sphingolipids can promote the progression of the cell cycle by regulating the activity of G1/S phase transcription factors, thereby accelerating the proliferation of gastric cancer cells (17), Currently, five S1P receptors (S1PR1–S1PR5) have been identified, each with unique tissue distribution and functions (**Table 1**; **Figure 3**). Furthermore, the role of sphingolipids in inducing apoptosis also provides a basis for their potential applications in tumor treatment; research has found that increased sphingolipids can induce tumor cells to enter an apoptotic state by affecting the cell cycle process, thereby inhibiting their proliferation (18). Sphingolipids effectively regulate cell proliferation by affecting the expression of factors that control the cell cycle’s progression. For instance, increases in sphingosine and ceramides are associated with cell cycle inhibition, as these molecules can inhibit the expression of cyclin D1 and CDK4, preventing cells from transitioning from G1 to S phase, thus inhibiting cell proliferation (19). Additionally, sphingolipids can also affect the progression of the cell cycle by regulating the activity of E2F transcription factors. Specifically, metabolic products of sphingolipids can inhibit the transcriptional activity of E2F by binding to it, further obstructing the advancement of the cell cycle (20).

**Table 1:** S1P receptor subtypes.

Receptor subtype	Primary distribution locations	Core functions
S1PR1	Endothelial cells, Lymphocytes, Cardiovascular system, Nervous system	Lymphocyte trafficking regulation, Angiogenesis, Endothelial barrier protection, Heart rate regulation
S1PR2	Nervous system, Kidney, Liver , Smooth muscle cells, Osteoblasts	Vasoconstriction, Hearing maintenance, Fibrosis regulation, Tumor migration inhibition
S1PR3	Cardiovascular system, Lung, Spleen, Nervous system	Vasodilation, Cardioprotection, Inflammatory response, Pro-tumor angiogenesis
S1PR4	Lymphocytes, Dendritic cells, Hematopoietic tissues	Immune cell differentiation, Inflammation regulation, Platelet activation
S1PR5	Central nervous system (CNS) - oligodendrocytes, NK cells	Oligodendrocyte differentiation and myelination, NK cell migration regulation

**Figure 4:**
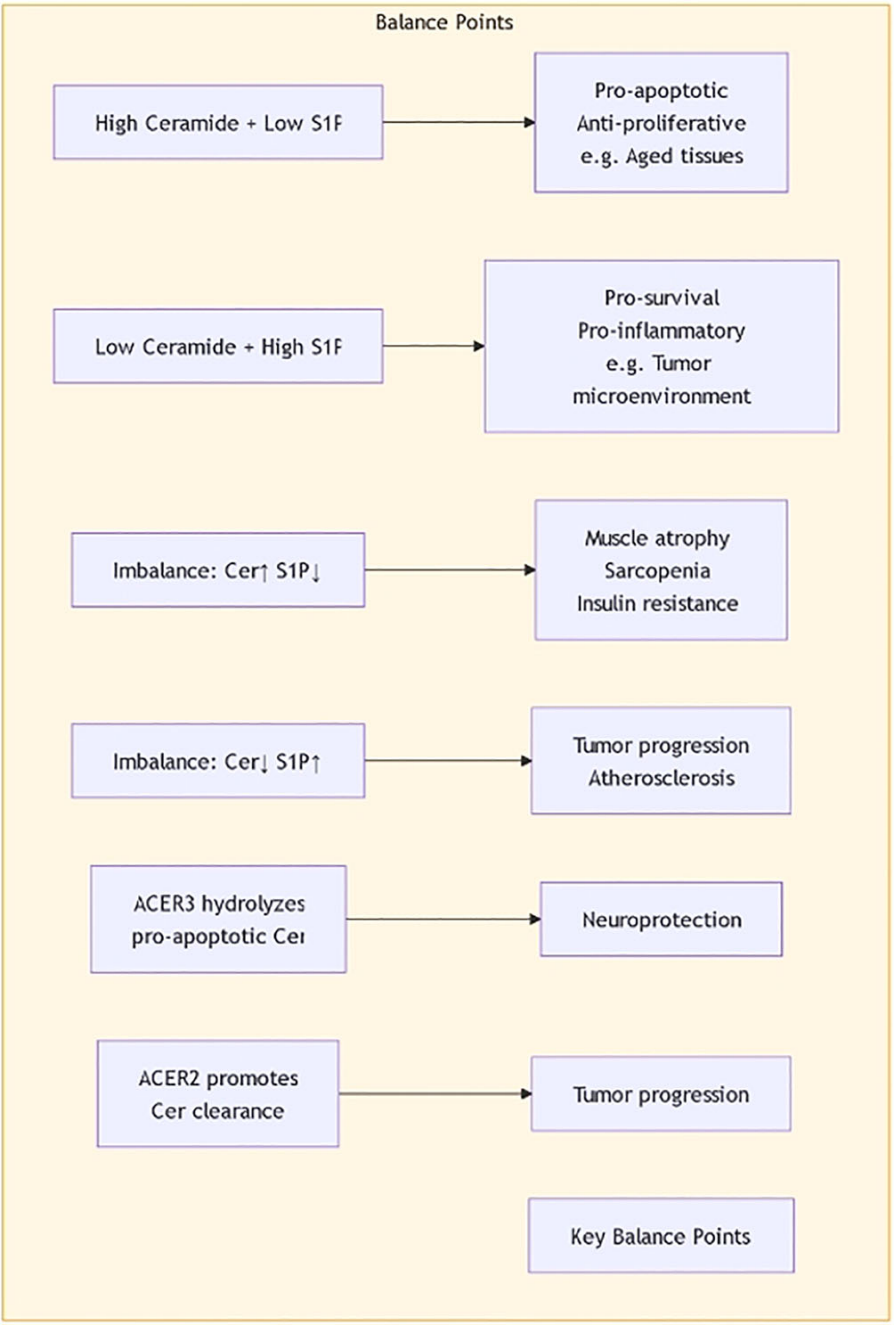
The balance of Ceramide and S1P.

The regulatory mechanisms of sphingolipid metabolism are complex, involving multiple signaling pathways and regulatory factors. Research shows that maintaining the balance between sphingolipid synthesis and degradation is essential for cellular functions. For example, ceramide synthases and sphingomyelinases are regulated by factors such as cellular energy status, oxygen levels, and extracellular growth factors. Recent studies indicate that Sphingosine-1-phosphate (S1P) plays a central role in regulating sphingolipid metabolism. S1P not only participates in cell proliferation and survival but also plays roles in inflammation and immune responses, affecting the metabolic balance of sphingolipids (**Figure 4**) (21, 22). Additionally, ORM family proteins have been identified as important factors regulating sphingolipid synthesis, maintaining sphingolipid homeostasis by modulating signaling between the endoplasmic reticulum and cell membrane. This regulatory mechanism shows significant biological relevance in various disease states (such as tumors, neurodegenerative diseases, etc.) (23, 24). By further studying the regulatory mechanisms of sphingolipid metabolism, new therapeutic strategies can be developed to address diseases associated with sphingolipid metabolic disorders.

### 2.2 Abnormal sphingolipid metabolism in gastric cancer

#### 2.2.1 Changes in sphingolipid metabolism in gastric cancer cells

Sphingolipids are a class of important lipid molecules that play key roles in the structure and function of cell membranes. Research has shown that there are notable changes in sphingolipid metabolism in gastric cancer cells, which may be closely related to the occurrence and development of cancer. The sphingolipid synthesis pathway in gastric cancer cells is regulated, leading to the accumulation of metabolic products such as sphingosine and ceramides, which are believed to be associated with biological processes such as cell proliferation, apoptosis, and migration. Abnormal sphingolipid metabolism may promote the survival and proliferation of cancer cells by affecting cell signaling pathways. Changes in sphingolipid metabolism may also be closely related to the microenvironment of gastric cancer, where the infiltration of tumor-associated macrophages and other immune cells may further influence the dynamic balance of sphingolipid metabolism. By further studying the changes in sphingolipid metabolism in gastric cancer cells, new ideas and strategies for targeted therapy can be provided (25–27).

The role of sphingolipids in cellular signaling pathways has garnered increasing attention, particularly regarding tumor cell proliferation and apoptosis. Studies have demonstrated that signaling molecules derived from sphingolipid metabolism, such as sphingosine-1-phosphate (S1P), can bind to S1P receptors on the cell surface, thereby activating downstream signaling cascades including the PI3K/Akt and MAPK pathways. (16). This activation subsequently modulates ERK activity, influencing cellular proliferation and apoptosis (28).Under normal physiological conditions, ceramide produced through sphingolipid metabolism induces apoptosis. However, during the initiation and progression of gastric cancer, tumor cells upregulate the activity of enzymes such as sphingolipid synthases to convert ceramide into sphingomyelin or sphingosine-1-phosphate, substances known for their anti-apoptotic properties. This metabolic shift inhibits apoptosis, thereby promoting the survival and proliferation of gastric cancer cells. Upon exposure to external stimuli such as chemotherapy or radiotherapy, the sphingolipid metabolic pathway is activated, leading to increased ceramide production and subsequent induction of apoptosis in gastric cancer cells. For instance, the chemotherapeutic agent paclitaxel has been shown to stimulate ceramide generation in gastric cancer cells, thereby facilitating apoptotic cell death. The activation of these signaling pathways is not only implicated in the proliferation and apoptosis of gastric cancer cells but may also be associated with tumor invasiveness and metastasis. Therefore, an in-depth investigation into the relationship between sphingolipids and cellular signaling pathways is crucial for elucidating their underlying mechanisms in the development and progression of gastric cancer, potentially offering novel targets and strategies for therapeutic intervention.

In gastric cancer cells, alterations in sphingolipid metabolism are closely associated with the differentiation status of the cells. Key enzymes in sphingolipid metabolism, such as sphingosine kinase and sphingosine-1-phosphate (S1P), exert significant effects on cellular proliferation and differentiation. Studies have revealed that S1P can modulate intracellular signaling pathways; for instance, the binding of S1P to its receptor S1P receptor 3 enhances Notch signaling, thereby increasing the population of gastric cancer stem cells. This process promotes the growth and differentiation of gastric cancer cells, ultimately impacting tumor progression and prognosis (29).

Increased sphingosine is associated with the upregulation of specific differentiation markers, including cell adhesion molecules and specific transcription factors, which play important roles in cell differentiation and migration (12). Furthermore, sphingolipids can also influence the expression of genes related to cell differentiation by regulating intracellular signaling pathways, such as the PI3K/Akt and MAPK pathways, thereby altering the biological behavior of cells (30).

Sphingolipids affect cell movement by modulating cell membrane fluidity and signal transmission, which are crucial in the metastatic process of gastric cancer cells. Research indicates that sphingolipid metabolites promote cell migration and invasion by influencing cytoskeletal reorganization. Increased sphingolipid modification enhances cell motility, facilitating the detachment of cancer cells from the primary tumor and their migration to other tissues (31). S1P can also enhance the migratory and invasive capabilities of gastric cancer cells by regulating cytoskeletal reorganization and the expression of cell adhesion molecules. For example, S1P has been shown to upregulate the expression of matrix metalloproteinases (MMPs) in gastric cancer cells, enabling the degradation of the extracellular matrix and thereby facilitating tumor metastasis. Sphingolipids are also crucial in cell signaling. They activate various pathways, such as PI3K/Akt and MAPK, which are known to promote tumor cell proliferation and metastasis (32).

The increase in sphingolipids leads to changes in cell membrane polarity, thereby affecting signal transmission and cytoskeletal reorganization (31). Additionally, the metabolic products of sphingolipids can promote cell movement by activating specific signaling pathways. For example, the increase in sphingosine is closely related to enhanced cell motility, indicating that the role of sphingolipids in cell migration should not be overlooked (33). Thus, investigating the relationship between sphingolipids and cell motility is essential for understanding the metastatic mechanisms in gastric cancer cells.

#### 2.2.2 Relationship between sphingolipid metabolism and prognosis in gastric cancer

Abnormal sphingolipid metabolism is significantly altered in gastric cancer cells and is closely linked to patient prognosis. Studies have shown that the expression levels of certain sphingolipid metabolism-related genes are significantly associated with the survival rates of gastric cancer patients. APOA1 mRNA and serum APOA1 protein are potential diagnostic and prognostic biomarkers for gastric cancer. Their expression levels are closely linked to clinical outcomes in patients (34). Additionally, machine learning methods have been used to identify key signatures of sphingolipid metabolism genes, which help define the immune microenvironment and prognosis of gastric cancer patients, offering new opportunities for personalized treatment (35). Thus, abnormalities in sphingolipid metabolism not only enhance our understanding of the biological mechanisms of gastric cancer but also provide critical evidence for clinical prognosis assessment and treatment strategy development. Through further research, sphingolipid metabolism may become a new target for gastric cancer treatment, providing new opportunities to improve patient survival rates and quality of life (36, 37).

### 2.3 Interaction between sphingolipids and immune cells

#### 2.3.1 Effects of sphingolipids on tumor-infiltrating immune cells

Sphingolipids regulate the functions of tumor-infiltrating immune cells (**Table 2**). Studies indicate that alterations in sphingolipid metabolism affect the polarization of tumor-associated macrophages (TAMs), which in turn influences tumor progression and immune evasion. The accumulation of sphingolipid amides and sphingosine is linked to an increase in M2 macrophages, which promote tumor growth and metastasis while inhibiting M1 macrophage activity; this shift is likely mediated by the regulation of cytokine secretion (38). Metabolic products of sphingolipids, such as sphingosine and ceramides, regulate T cell functions, influencing their proliferation and cytokine secretion. This indicates that sphingolipids are significant in both macrophage and T cell immune responses (31). Therefore, changes in sphingolipid metabolism may provide new targets for tumor immunotherapy.

**Table 2:** Sphingolipid metabolism modulates immune cell function and therapeutic targets in the tumor microenvironment.

Types of immune cells	Mechanism of action	Effects on the tumor	Associated metabolic products/pathways	Prospective therapeutic targets
Tumor-Associated Macrophages	·Ceramide induces M2 polarization ·Activates the PI3K/Akt pathway to promote the secretion of pro-inflammatory cytokines	·Promotes tumor invasion ·Accelerates angiogenesis ·Mediates the formation of an immunosuppressive microenvironment	Ceramide, Sphingosine, PI3K/Akt pathway	Sphingosine kinase inhibitors (such as SKI-II)
T cells	·Sphingolipid metabolites inhibit CD8^+^ T cell proliferation ·Downregulate IFN-γ secretion via the MAPK pathway	·Impairs tumor immune surveillance ·Promotes liver metastasis ·Reduce chemosensitivity	S1P, MAPK pathway	S1P receptor antagonists (such as FTY720)
Regulatory T cells (Tregs)	·Sphingolipid metabolism enhances the ·immunosuppressive function of Tregs via the PP2A pathway	·Inhibits anti-tumor immune response ·Promotes peritoneal dissemination	Ceramide, PP2A phosphatase system	Ceramide analogs (such as C6-ceramide)
Dendritic cells (DCs)	·Sphingolipid dysregulation leads to impaired maturation of dendritic cells (DCs) ·Reduces tumor antigen-presenting capacity	·Impairs immune activation function ·Promotes lymph node metastasis	Sphingosine-1-phosphate lyase (SPL)	SPL activators (such as THI)

#### 2.3.2 Role of sphingolipids in regulating the tumor microenvironment

The role of sphingolipids in the tumor microenvironment is not limited to affecting immune cell behavior; it also involves interactions between tumor cells and stromal cells. S1P can promote the secretion of angiogenic factors such as vascular endothelial growth factor (VEGF), thereby inducing tumor angiogenesis and providing nutritional and oxygen support for the growth and metastasis of gastric cancer cells. Additionally, S1P facilitates the formation of tumor vasculature by regulating the migration and proliferation of endothelial cells. Studies have shown that sphingolipids regulate intercellular signaling and alter the characteristics of the tumor microenvironment. For instance, sphingolipid metabolites can influence the expression of chemokines such as CXCL2, thereby promoting the infiltration of tumor-associated macrophages, which in turn enhances tumor growth and metastatic potential (39). Moreover, sphingolipid metabolism is closely linked to the metabolic reprogramming of tumor cells; the accumulation of sphingolipids may drive adaptive changes that improve tumor cell survival and growth within the microenvironment (5). Therefore, elucidating the mechanistic role of sphingolipids in the tumor microenvironment may contribute to the development of novel therapeutic strategies aimed at improving prognosis and treatment responsiveness in cancer patients.

### 2.4 Sphingolipid metabolism and immune evasion mechanisms

#### 2.4.1 How sphingolipids promote immune evasion of tumor cells

Sphingolipids are important bioactive lipids. Recent studies indicate that their metabolism is crucial for the immune evasion of tumor cells. Tumor cells effectively suppress the host’s anti-tumor immune response by changing how sphingolipids are metabolized. Tumor cells can promote the formation of an immunosuppressive microenvironment by increasing the synthesis of sphingosine and ceramides, thereby inhibiting T cell activity and proliferation. This mechanism not only reduces the chance of tumor cells being recognized by the immune system but may also enhance their immune evasion by inducing regulatory T cells (Tregs). Additionally, sphingolipids can change how tumor cells respond to immune cells. They do this by affecting cell membrane fluidity and signaling pathways, which promotes tumor growth and metastasis (12). The S1P signaling pathway can modulate the function of immune cells within the tumor microenvironment, such as by suppressing the activity of natural killer (NK) cells and cytotoxic T lymphocytes (CTLs). This immunosuppressive effect enables gastric cancer cells to evade immune surveillance and destruction, thereby facilitating tumor growth and progression. Studies suggest that targeting sphingolipid metabolism may provide new approaches for cancer immunotherapy (40).

#### 2.4.2 Relationship between immune checkpoints and sphingolipid metabolism

Immune checkpoint activation is crucial for tumor immune evasion, and the interaction between sphingolipid metabolism and immune checkpoints is receiving increasing attention. Metabolic products of sphingolipids, particularly ceramide, can upregulate PD-L1 expression. This process enhances the immunosuppressive effects of tumor cells on the immune system (41). By regulating immune checkpoint expression, tumor cells can effectively evade T cell attacks, resulting in immune tolerance. Additionally, abnormalities in sphingolipid metabolism can impair immune cell functions, such as dendritic cell maturation and antigen presentation, which weakens the anti-tumor immune response (42). Gastric cancer cells can develop resistance to chemotherapeutic agents through alterations in the sphingolipid metabolic pathway. For example, the overexpression of sphingomyelin synthase allows cancer cells to convert pro-apoptotic ceramide into sphingomyelin, thereby reducing ceramide-induced apoptosis and diminishing the efficacy of chemotherapy. In addition, certain metabolites generated during sphingolipid metabolism can contribute to chemoresistance by modulating intracellular signaling pathways, further promoting the survival of gastric cancer cells in the presence of anticancer drugs. Therefore, in-depth investigation into the relationship between sphingolipid metabolism and immune checkpoints may facilitate the development of novel immunotherapeutic strategies aimed at overcoming tumor immune evasion and resistance to conventional chemotherapeutic agents (43).

### 2.5 Future research directions and clinical application prospects

#### 2.5.1 Potential of sphingolipid metabolism as a therapeutic target

Sphingolipid metabolism significantly contributes to various diseases, particularly cancer, cardiovascular diseases, and metabolic syndrome. Studies indicate that sphingolipid molecules, including ceramides and sphingomyelins, play a role in biological processes like cell proliferation, apoptosis, and inflammation, thus making them potential therapeutic targets. Abnormal sphingolipid metabolism is a key factor in atherosclerosis, and interventions targeting this pathway are expected to improve its pathological conditions (44). Additionally, the role of sphingolipid metabolism in cancer has also received widespread attention, with research finding that its regulatory role in the tumor microenvironment may influence immune evasion and tumor resistance, providing a theoretical basis for developing new anti-tumor strategies (12). Thus, future research should investigate how to develop and apply sphingolipid metabolism regulators to enhance patient prognosis and quality of life through targeted therapies.

#### 2.5.2 Prospects for combining sphingolipid metabolism with immunotherapy

The relationship between sphingolipid metabolism and the immune system offers fresh insights for improving immunotherapy. Research indicates that sphingolipid molecules function within tumor cells and also affect the tumor immune microenvironment. They do this by regulating immune cell functions. For example, ceramides can inhibit T cell activation and proliferation, thereby promoting tumor immune evasion (45). Therefore, regulating sphingolipid metabolism alongside immunotherapy may enhance its effectiveness. F Future research should aim to boost the anti-tumor activity of immune cells by adjusting sphingolipid metabolism, particularly in patients who are resistant to current immunotherapies. Moreover, sphingolipid metabolism-related biomarkers are expected to be used to predict the efficacy of immunotherapy, thereby achieving personalized treatment strategies (46). In summary, the combination of sphingolipid metabolism and immunotherapy provides broad prospects for future clinical applications, warranting in-depth exploration.

## 3 Conclusion

This review highlights the significance of sphingolipid metabolism in the immune microenvironment of gastric cancer and its regulatory mechanisms, stressing its potential role in tumor development. Research shows that sphingolipids and their metabolic products play a role in building cell membranes. Additionally, they affect the tumor microenvironment by regulating cell signaling, inflammatory responses, and immune cell functions. These findings provide new perspectives for understanding the pathogenesis of gastric cancer and suggest that sphingolipid metabolism may become a new target for gastric cancer treatment.

However, there are still some limitations in current research, mainly reflected in small sample sizes, singular experimental models, and a lack of long-term follow-up data. Many studies focus on basic scientific exploration but lack translational research for clinical applications. Therefore, future research should focus on multi-center and large-scale clinical trials to verify the true role and clinical significance of sphingolipid metabolism in gastric cancer patients. Additionally, combining multi-omics technologies to explore the interactions between sphingolipid metabolism and other metabolic pathways and immune mechanisms will help comprehensively understand its complex role in gastric cancer.

Regarding treatment prospects for gastric cancer, it is essential to consider the potential of targeting sphingolipid metabolism as a new therapeutic approach. By targeting the regulation of sphingolipid metabolism, it may not only inhibit the growth of tumor cells but also improve the tumor microenvironment and enhance immune responses, thereby improving treatment outcomes. Future research should explore the application potential of different types of sphingolipid metabolism inhibitors and regulators in gastric cancer treatment, combining traditional therapeutic methods to develop comprehensive treatment plans aimed at improving patient prognosis.

In conclusion, sphingolipid metabolism holds considerable scientific value and promising clinical applications in gastric cancer research, highlighting the need for further investigation. We look forward to future studies filling current knowledge gaps and advancing this field to provide more effective treatment strategies for gastric cancer patients.
